# Inconsistent selection of outcomes and measurement devices found in shoulder arthroplasty research: An analysis of studies on ClinicalTrials.gov

**DOI:** 10.1371/journal.pone.0187865

**Published:** 2017-11-10

**Authors:** Matthew Thomas Sims, Byron Nice Detweiler, Jared Thomas Scott, Benjamin McKinnley Howard, Grant Richard Detten, Matt Vassar

**Affiliations:** Oklahoma State University Center for Health Sciences—Tulsa, OK, United States of America; University of Memphis, UNITED STATES

## Abstract

**Introduction:**

Recent evidence suggests a lack of standardization of shoulder arthroplasty outcomes. This issue is a limiting factor in systematic reviews. Core outcome set (COS) methodology could address this problem by delineating a minimum set of outcomes for measurement in all shoulder arthroplasty trials.

**Methods:**

A ClinicalTrials.gov search yielded 114 results. Eligible trials were coded on the following characteristics: study status, study type, arthroplasty type, sample size, measured outcomes, outcome measurement device, specific metric of measurement, method of aggregation, outcome classification, and adverse events.

**Results:**

Sixty-six trials underwent data abstraction and data synthesis. Following abstraction, 383 shoulder arthroplasty outcomes were organized into 11 outcome domains. The most commonly reported outcomes were shoulder outcome score (n = 58), pain (n = 33), and quality of life (n = 15). The most common measurement devices were the Constant-Murley Shoulder Outcome Score (n = 38) and American Shoulder and Elbow Surgeons Shoulder Score (n = 33). Temporal patterns of outcome use was also found.

**Conclusion:**

Our study suggests the need for greater standardization of outcomes and instruments. The lack of consistency across trials indicates that developing a core outcome set for shoulder arthroplasty trials would be worthwhile. Such standardization would allow for more effective comparison across studies in systematic reviews, while at the same time consider important outcomes that may be underrepresented otherwise. This review of outcomes provides an evidence-based foundation for the development of a COS for shoulder arthroplasty.

## 1 Introduction

Orthopedic shoulder pathology from age-related complications is increasing, due in part to longer lifespans. Osteoarthritis and rotator cuff disease are two degenerative conditions most commonly identified as causing pain and disability in the aging population [1]. While many treatments exist for initial stages of degenerative shoulder diseases, three interventions are most common for treatment of progressive to severe osteoarthritis. *Total shoulder arthroplasty* (TSA)—replacement of the humeral head and prosthetic resurfacing of the glenoid—is considered the gold standard treatment due to its reliable pain relief, predictable improvement of function, and enhanced quality of life [2–4]. *Hemiarthroplasty* (HA), which involves replacing the humeral head alone [1], and *reverse shoulder arthroplasty* (RSA), a modified TSA where the semi-circumference ball is implanted in the glenoid and a stem with a concave polyethylene cap implanted in the humerus, are also viable treatments for advanced shoulder disease. Exponential increases in use of these interventions warrant further study to better understand their efficacy, surgical indications, and potential complications [5–7]. Unfortunately, useful information on these aspects of shoulder arthroplasty is limited, partially due to methodological issues associated with the reported studies. For instance, a Cochrane systematic review of these surgeries for shoulder osteoarthritis was inconclusive, in part, because the primary studies comprising the review did not measure outcomes aligned with the research questions [8]. In fact, most outcomes important to the systematic reviewers were measured in only a single study. Another recent review of arthroscopy following shoulder arthroplasty noted significant heterogeneity in outcome reporting among primary studies which limited the reviewers’ ability to perform a quantitative synthesis of outcomes [9]. Standardization of outcomes for shoulder arthroplasty studies would help overcome limitations reported in previous systematic reviews and allow for more conclusive evaluations of efficacy.

Core outcome set (COS) methodology could address this problem using consensus approaches involving trialists, systematic reviewers, funders, patients, and other research stakeholders to derive a minimum set of outcomes for measurement across shoulder arthroplasty studies [10–12]. In conjunction with COS development, core outcome measurement sets establish the instruments that should be administered for outcome measurement [13]. For example, there are currently several shoulder measures available: the American Shoulder and Elbow Surgeons Society Standardized Shoulder Assessment Form (ASES), the Constant-Murley Shoulder Outcome Score, Disabilities of the Arm, Shoulder, and Hand Questionnaire (DASH), the *Quick*DASH, L’Insalata Shoulder Rating Questionnaire, Simple Shoulder Test (SST), the Oxford Shoulder Score (OSS), the Shoulder Disability Questionnaire (SDQ), and the Western Ontario Shoulder Instability Index (WOSI), among others [14,15]. Whether condition-specific symptoms should be limited to movement-related shoulder functions or more generally to include broader aspects of functioning (e.g., leisure activities or work) remains a source of debate [16] and the diversity of items and domains comprising such measures may impede meta-analytic investigations. A standardized recommendation for evaluation of shoulder function would improve the ability to synthesize evidence across studies. Given the impressive growth of TSA, HA, and RSA procedures, there is a need for continued evaluation of their efficacy and for determining whether an increased standardization of outcomes is necessary. Here, we conduct an analysis of shoulder arthroplasty intervention studies registered on ClinicalTrials.gov to elucidate the diversity of methodologies and outcomes reported. The objective of this study is to provide an evidence-based foundation for the development of a COS for shoulder arthroplasty.

## 2 Methods

We conducted an analysis of studies catalogued in ClinicalTrials.gov to examine outcomes reported in registered orthopedic surgery clinical trials. This study did not meet the regulatory definition of human subject research as defined in 45 CFR 46.102(d) and (f) of the Department of Health and Human Services’ Code of Federal Regulations [17] and, therefore, was not subject to Institutional Review Board oversight. We consulted Li et al [18], the Cochrane Handbook for Systematic Reviews of Interventions [19], and the National Academies of Science, Engineering, and Medicine’s (formally the Institute of Medicine) Standards for Systematic Reviews [20] for best practices in data collection and management for systematic reviews as we developed our methodology. To adhere to best practices in reporting, we applied relevant PRISMA guidelines [21] (Checklist items 1–3, 5–11, 13, 16–18, 20, 23, 24, 26, 27) since our study involved the synthesis of multiple registered trials. We applied SAMPL guidelines [22] for reporting descriptive statistics. This study was registered with the Core Outcome Measurement in Effectiveness Trials (COMET) Initiative (http://www.comet-initiative.org/studies/details/812?result=true). Data from this study is publicly available on figshare (https://dx.doi.org/10.6084/m9.figshare.3464831.v2).

### 2.1 Eligibility criteria for considering studies for this review

Primary studies registered in ClinicalTrials.gov between 2005 and 2015 in which shoulder arthroplasty (including total shoulder arthroplasty, reverse shoulder arthroplasty, hemiarthroplasty, and glenoid resurfacing) was used as an intervention were eligible for this review. For this study, both open (not yet recruiting, recruiting) and closed (active, not recruiting; completed; terminated; suspended; withdrawn; enrolling by invitation) trials were eligible for inclusion. Randomized and non-randomized clinical trials as well as observational studies were included since these study designs may be registered on ClinicalTrials.gov [23]. We used the following definitions to classify study types. A clinical trial (National Institutes of Health definition) was defined as “a research study in which one or more human subjects are prospectively assigned to one or more interventions (which may include placebo or other control) to evaluate the effects of those interventions on health-related biomedical or behavioral outcomes.” An observational study was defined as “a biomedical or behavioral research study of human subjects designed to assess risk factors for disease development or progression, assess natural history of risk factors or disease, identify variations based on geographic or personal characteristics (such as race/ethnicity or gender), track temporal trends, or describe patterns of clinical care and treatment in absence of specific study-mandated interventions” [24].

### 2.2.1 Search strategy for identifying relevant studies

We consulted a research librarian to conduct a search for clinical trials registered on ClinicalTrials.gov that examined shoulder arthroplasty interventions reported in orthopedic surgery literature. ClinicalTrials.gov was searched in order to identify unpublished or ongoing trials. We used registered trials to minimize the possibility of selective outcome reporting bias^30^ and to better understand the outcomes reported in current orthopedic clinical trials. This search was narrowed for four common arthroplasty shoulder procedures: total shoulder arthroplasty (TSA), reverse shoulder arthroplasty (RSA), hemiarthroplasty (HA), and glenoid resurfacing; however, we did not impose a limiter for language or restrict the search by journal. The final search string is as follows: Shoulder AND (Surg* OR operat* OR arthroplasty OR hemiarthroplasty OR (joint* AND replace*) OR debride OR debridement OR debrided OR (surface AND (replace OR replacement OR replaced)) OR resurface OR resurfaced OR resurfacing) | received from 01/01/2005 to 12/31/2016. The search was performed on June 30, 2017.

### 2.2.2 Study selection and data collection

**Fig 1:**
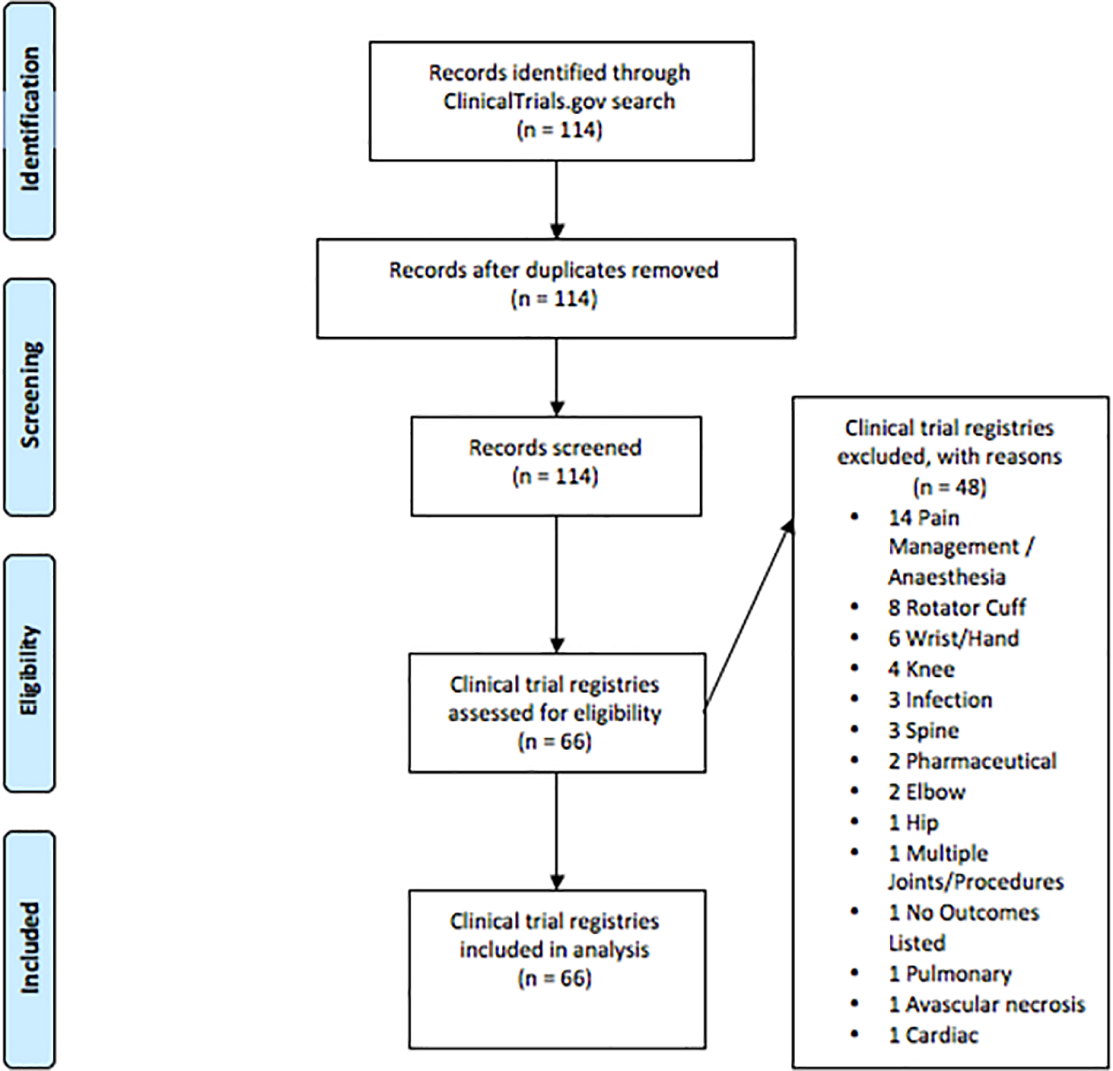
PRISMA flowchart. PRISMA flowchart displaying the search results along with the included and excluded studies.

Four authors (MTS, JTS, BMH, and GRD) equally divided the studies among one another and independently screened all of the studies for eligibility. To be eligible, a study must have reported the use of shoulder arthroplasty as an intervention. We included total, hemi-, and reverse arthroplasty as well as glenoid resurfacing; hence, arthroscopic studies were excluded from analysis. Studies must also have been registered on ClinicalTrials.gov between 2000 and 2016. We included both observational and interventional studies, as both commonly report primary and secondary outcomes in ClinicalTrials.gov. After the initial screening was completed, a second screening was performed by an author (BND) who was blinded from previous screening results. Discrepancies in screening were resolved by discussion between BND and the other authors. Final exclusions are outlined in the PRISMA flow diagram (Fig 1).

An abstraction manual was designed after consulting several sources [25–30] to ensure data abstraction was consistently and accurately performed by authors. Authors participated in a series of meetings to apply the abstraction manual to a subset of 15 studies as a pilot test before launch. During these meetings, authors abstracted data elements by reviewing each study, discussing data elements, and reaching agreement on changes to the abstraction manual. Refinements were made based on pilot feedback and a final manual was produced. Data elements included:

- sponsor(s), title of the article;
- start date of trial (year);
- study status (not yet recruiting; recruiting; active, not recruiting; completed; terminated; suspended; withdrawn; enrolling by invitation)
- study type (interventional, observational, etc.);
- type of arthroplasty (TSA, RSA, HA, glenoid resurfacing, other);
- sample size;
- measured outcomes;
- outcome measurement device;
- specific metric of measurement (value at a time point, change from baseline, time to event, unclear);
- method of aggregation (mean, median, percent/proportion, absolute number, unclear);
- outcome classification (primary, secondary, other, unclear);
- whether the outcome was considered a side effect/harmful.

The registered studies meeting inclusion criteria were then equally divided for data abstraction among four authors (MTS, GRD, JTS, and BMH). Working in pairs, authors first abstracted data elements from articles in their set and then validated the abstracted data of their partner. Any discrepancies in data abstraction were settled by discussion between the pair, or when necessary, by adjudication with the blinded author (BND) to ensure the accuracy and integrity of this study.

### 2.3 Definition and classification of measured outcomes

We defined an outcome as the exact word-for-word terms (presented as either a primary or secondary outcome) in a trial for any clinical endpoint, or physiological, metabolic, or mortality event measured by clinicians or researchers [26]. Eleven outcome domains were determined based on the distribution of outcomes within this study and previously defined domains by Page et al [28]. Outcomes were classified under the following outcome domains: Adverse Events, Function/Disability, Global Assessment of Treatment Success, Health Related Quality of Life (HRQoL), Orthopedic Tests, Other, Pain, Radiologic Evaluation, Range of Motion (ROM), Strength, and Survival. Individual outcomes were distributed into each of these categories during the coding process. In order to decrease heterogeneity of reported outcomes, authors determined standardized terminology for each outcome.

### 2.4 Statistical analysis

Results were summarized using frequencies and percentages for binary outcomes, and medians and interquartile ranges (IRQs) for continuous outcomes. Locally weighted scatterplot smoothing (nonparametric regression method) was used to smooth the scatterplots of outcome domain use over time [28]. Our final scatterplot data is available on figshare (https://dx.doi.org/10.6084/m9.figshare.3464831.v2). Descriptive statistics were used to summarize data and all analyses were conducted using STATA 13.1 (College Station, TX).

## 3 Results

A total of 114 clinical trials were identified on ClinicalTrials.gov. Forty-eight studies were excluded after failing to meet inclusion criteria (Fig 1). A final sample size of 66 trials underwent data abstraction and was included in the final data synthesis. Clinical trials included within this study started their research between 2000 and 2016, as summarized in Table 1.

**Table 1:** Characteristics of included studies (Updated to Reflect 2016 Data).

Characteristics	Number (%) of trials (n = 66)
*Start Date of Trial*	
2000–2004	6 (9.1)
2005–2008	13 (19.7)
2009–2012	20 (30.3)
2013–2016	27 (40.9)
*Phase of Trial*	
Active, Not Recruiting	10 (15.2)
Completed	14 (21.2)
Enrolling by Invitation	6 (9.1)
Not yet Recruiting	3 (4.5)
Recruiting	18 (27.3)
Suspended	1 (1.5)
Terminated	4 (6.1)
Unknown	9 (13.6)
Withdrawn	1 (1.5)
*Type of Trial*	
Interventional	37 (56.1)
Observational	29 (43.9)
*Procedure Frequency*	
Hemiarthroplasty (HA)	15 (16.9)
Total Shoulder Arthroplasty (TSA)	37 (41.6)
Reverse Shoulder Arthroplasty (RSA)	24 (27)
Glenoid Resurfacing	11 (12.3)
Other	2 (2.2)

### 3.1 Summary of shoulder arthroplasty trials characteristics

Nearly half of the studies were comprised of “Completed” (14/66, 21.2%) and “Recruiting” (18/66, 27.3%) studies. “Active, not recruiting” and “Unknown” trials each accounted for 10 and 9, respectively, (19/66, 28.8%) of the remaining trials (Table 1). Of the 66 studies, 37 were listed as interventional (37/66, 56.1%) and 29 were listed as observational (29/66, 43.9%). The most frequently reported shoulder arthroplastic procedure was TSA (37/66, 41.6%). RSA (24/66, 27%), HA (15/66, 16.9%), and glenoid resurfacing (11/66, 12.3%) were also commonly reported shoulder arthroplasties (Table 1).

### 3.2 Shoulder arthroplasty outcomes and domain categories

Following data abstraction, 383 shoulder arthroplasty outcomes were organized into 11 different outcome domains. The standardized outcomes, measurement devices and specific metrics were summarized and organized into domain categories, as displayed in Table 2.

**Table 2:** Domain categories and reported outcomes, device, and metric within each domain (Updated to Reflect 2016 Data).

Domains (n = 11)	Outcomes (n = 383)	Measurement Devices	Specific Metric
**Adverse Events** **(n = 25)**	Adverse events (7) Device associated adverse events (3) Biceps rupture (1) Device migration (1) Intraoperative bleeding (1) Intraoperative neurovascular injury (1) Intraoperative prosthetic fracture (1) Lack of unanticipated device related serious adverse events (1) Osteolysis (1) Postoperative bleeding (1) Postoperative infection (1) Postoperative instability (1) Procedure associated adverse events (2) Revision complications (1)	Frequency (19) Unspecified (3)	Value at a time point (20) Unspecified (2)
**Function/Disability** **(n = 16)**	Function (15) Function/disability (1)	ASES (4) SST (4) Constant (3) SANE (2) Clinical outcome comparison (1) Unspecified (1) VAS (1)	Value at a time point (13) Change from baseline (3)
**Global Assessment of Treatment Success** **(n = 60)**	Shoulder outcome score (58) Effectiveness (1) Impact of arm length difference on patient reported outcome (1)	Constant (2) ASES (19) Oxford (5) PENN (3) DASH (3) QuickDASH (3) SSV (2) Modified Constant (1) Neer’s limited goals (1) SANE (1) SPADI (1) UCLA (1)	Value at a time point (42) Change from baseline (12)
**Health Related Quality of Life** **(n = 68)**	Quality of life (15) Multidimensional aspects of health (8) Patient satisfaction (9) Activities of daily living (4) Disease or joint specific measure (4) General health component (2) Patient objective data (2) Patient subjective data (2) Anxiety/depression (1) Mental component summary (1) Mental health component (1) Mobility (1) Personal dependency status (1) Physical component summary (1) Physical function (1) Role emotional (1) Role physical (1) Self-care (2) Social function (1) Usual activities (1) VAS (1) Vitality (1) Willingness to have surgery performed again (1)	EQ5D (11) SF-36 (10) WOOS (8) SF-12 (6) Unspecified (6) ASES (2) EQ5D5L (2) Patient assessment forms (2) VAS (2) 15D (2) ADLER (1) SPADI (1) Unspecified (1) PENN (1) 4-point rating (1) Constant (1) DASH (1) Quality of life survey (1) SST (1)	Value at a time point (39) Change from baseline (20) Unspecified (1)
**Orthopedic Tests** **(n = 7)**	Test specific outcomes (4) Integrity & function of subscapularis tendon (2) Internal rotation extension (1)	Abdominal compression test (2) Lift off test (2) Hornblower’s test (1) Speed’s test (1) Yergason’s test (1)	Change from baseline (5) Value at a time point (2)
**Other** **(n = 23)**	Cost association (1) Health economics (1) Recovery time (1) Surgical time (1)	Unspecified (3) Health resource utilization instrument (1)	Value at a time point (4)
**Pain** **(n = 35)**	Pain (33) Pain at rest (1) Pain with active motion (1) Pain/discomfort (2) Pain/weakness (1) Preoperative pain (1)	VAS (10) ASES (6) NRS (3) Unspecified (3) SANE (2) Clinical outcome comparison (1) Constant (2) SF-36 (1) SPADI (1) EQ5D (1) PENN (1) Likert scale (1)	Value at a time point (21) Change from baseline (6)
**Radiologic Evaluation** **(n = 79)**	Acromiohumeral interval (1) Actual versus optimal glenosphere position (1) Actual versus predicted scapular notching (1) Bone density around the prosthesis (1) Bony apposition (2) Bony integration of the component (1) Clinical outcome comparison (1) Component loosening (3) Component position (1) Coracoid to glenohumeral joint distance (1) Coracoid to tuberosity distance (1) Correlation between bone density and prosthesis migration (1) Cumulative incidence of migration, radiolucency, osteolysis, and bone wear (1) Decreased component loosening (2) Decreased radiolucent lines (2) Determine if the use of autologous bone graft around the anchor-peg glenoid prosthesis correlate with bony apposition (1) Determine if the use of autologous bone graft around the anchor-peg glenoid prosthesis correlate with decreased radiolucent lines and component loosening (1) Determine if the use of autologous bone graft around the anchor-peg glenoid prosthesis correlate with functional outcomes (1) Difference in component migration between conventional and lateralized glenoid components (1) Evidence of movement or pending failure (1) Fixation to bone/early migration of the implants (1) Function (2) Glenoid component migration (1) Glenoid component position (3) Glenoid erosion (3) Glenoid status (2) Head to tuberosity distance (1) Humeral congruity (1) Humeral cortical thickness (1) Humeral stem position–valgus/varus (1) Humeral subluxation (1) Lateralization index (3) Location and placement of HRA device (1) Radiographic evaluation (3) Migration between cemented and press-fit RTSA humeral components (1) Migration of resurfacing prosthesis (1) No evidence of device failure (1) Postoperative clinical results (1) Qualitative documentation of surgical steps (1) Radiographic failure (1) Radiolucent lines (5) Scapular notching (1) Subacromial space (3) Subsidence (5) Tuberosity thinning (3) X-Rays (3)	Radiograph (21) 3D CT (2) Unspecified (13) CT (12) Millimeters (2) Frequency (1) Radiostereometric analysis (3) Degrees (1) Plain radiograph (2) MRI (7) X-Ray (9) Modified Tingart & Al method (1) Intraoperative photographs (1)	Value at a time point (61) Change from baseline (13) Unspecified (1)
**Range of Motion** **(n = 38)**	Range of motion (11) External rotation (4) Internal rotation (3) Active external rotation (2) Flexion (2) Passive external rotation (2) Active abduction (1) Active flexion (1) Active internal rotation (1) Active range of motion (1) Passive abduction (1) Passive flexion (1) Passive internal rotation (1) Passive range of motion (1) Postoperative clinical results (1) Scaption (1)	Constant (10) Unspecified (17) Goniometer (4) Range of motion (1) ASES (1) Degrees (1)	Change from baseline (21) Value at a time point (13)
**Strength** **(n = 16)**	External rotation strength (2) Flexion strength (2) Internal rotation strength (2) Strength (2) External abduction strength (1) Scapula abduction strength (2) Thumb down abduction strength (1)	Pounds (5) Lafayette manual muscle testing system (4) Iso-force machine (2) Unspecified (1)	Value at a time point (10) Change from baseline (2)
**Survival** **(n = 16)**	Implant survival (11) Revision/reoperation (3) Device success rate (1) Time to first revision (1)	Frequency (6) Kaplan-Meier (5) Unspecified (5)	Time to event (11) Value at a time point (4) Unspecified (1)

The Radiologic Evaluation domain contained the greatest number of outcomes (n = 79) followed by the HRQoL (n = 68) and Global Assessment of Treatment Success (n = 60) domains (Table 2). In terms of outcome reporting, the Radiologic Evaluation domain contained a large number of unique outcomes that were measured in a few studies. The Global Assessment of Treatment Success domain contained the most commonly reported outcome, shoulder outcome score (n = 58). Pain (n = 33), quality of life (n = 15), function (n = 15), ROM (n = 11) and implant survival (n = 11) were also frequently reported outcomes (Table 3). Across all domains, 61 outcomes had an unspecified measurement device. The most common measurement devices were the Constant-Murley Shoulder Outcome Score (n = 38), American Shoulder and Elbow Surgeons (ASES) Shoulder Score (n = 33), and frequency counts (such as number of adverse events or revisions) (n = 30) (Table 2).

**Table 3:** Outcomes reported by frequency of measurements (Updated to Reflect 2016 Data).

**Outcomes reported in >5 studies**			
Adverse events	Function	Implant Survival	Multidimensional aspects of health
Pain	Patient satisfaction	Range of motion	Shoulder outcome score
Quality of life			
**Outcomes reported in 2–5 studies**			
Active external rotation	Activities of daily living	Bony apposition	Component loosening
Decreased component loosening	Decreased radiolucent lines	Device associated adverse events	Disease or joint specific measure
External rotation	External rotation strength	Flexion	Flexion strength
General health component	Glenoid component position	Glenoid erosion	Glenoid status
Integrity & function of subscapular tendon	Internal rotation	Internal rotation strength	Lateralization index
Passive external rotation	Patient objective data	Patient subjective data	Postoperative clinical results
Radiographic evaluation	Radiolucent lines	Revision/reoperation	Scapula abduction strength
Strength	Subacromial space	Subsidence	Test specific outcomes
Tuberosity thinning	X-Rays		
**Outcomes reported only once**			
Acromiohumeral interval	Active abduction	Active flexion	Active internal rotation
Active range of motion	Actual versus optimal glenosphere position	Actual versus predicted scapular notching	Anxiety/depression
Biceps rupture	Bone density around the prosthesis	Bony integration of the component	Clinical outcome comparison
Component position	Coracoid to glenohumeral joint distance	Coracoid to tuberosity distance	Correlation between bone density and prosthesis migration
Cost association	Cumulative incidence of migration, radiolucency, osteolysis, and bone wear	Determine if the use of autologous bone graft around the anchor-peg glenoid prosthesis correlate with decreased radiolucent lines and component loosening	Determine if the use of autologous bone graft around the anchor-peg glenoid prosthesis correlate with functional outcomes
Determine if the use of autologous bone graft around the anchor-peg glenoid prosthesis correlate with bony apposition	Device migration	Device success rate	Difference in component migration between conventional and lateralized glenoid components
Effectiveness	Evidence of movement or pending failure	External abduction strength	Fixation to bone/early migration of the implants
Function/disability	Glenoid component migration	Head to tuberosity distance	Health economics
Humeral congruity	Humeral cortical thickness	Humeral stem position-valgus/varus	Humeral subluxation
Impact of arm length difference on patient reported outcome	Internal rotation extension	Intraoperative bleeding	Intraoperative neurovascular injury
Intraoperative prosthetic fracture	Lack of unanticipated device related serious adverse events	Location and placement of HRA device	Mental component summary
Mental health component	Migration between cemented and press-fit RTSA humeral components	Migration of resurfacing prosthesis	Mobility
No evidence of device failure	Osteolysis	Pain at rest	Pain with active motion
Pain/discomfort	Pain/weakness	Passive abduction	Passive flexion
Passive internal rotation	Passive range of motion	Personal dependency status	Physical component summary
Physical function	Postoperative bleeding	Postoperative infection	Postoperative instability
Preoperative pain	Procedure associated adverse events	Qualitative documentation of surgical steps	Radiographic failures
Recovery time	Revision complications	Role emotional	Role physical
Scaption	Scapular notching	Self-care	Social function
Surgical time	Thumb down abduction strength	Time to first revision	Usual activities
Visual analog scale	Vitality	Willingness to have surgery performed again	

There was a mean of six outcomes reported per study, with a range between one and thirty-seven outcomes reported per study. In each trial registry, the outcomes received a classification of primary, secondary, other, or unspecified. Of the 383 reported outcomes, 68.7% (263/383) were classified as secondary outcomes and the remaining were predominantly primary outcomes (120/383, 31.3%).

### 3.3 Frequency of outcome domains over time

**Fig 2:**
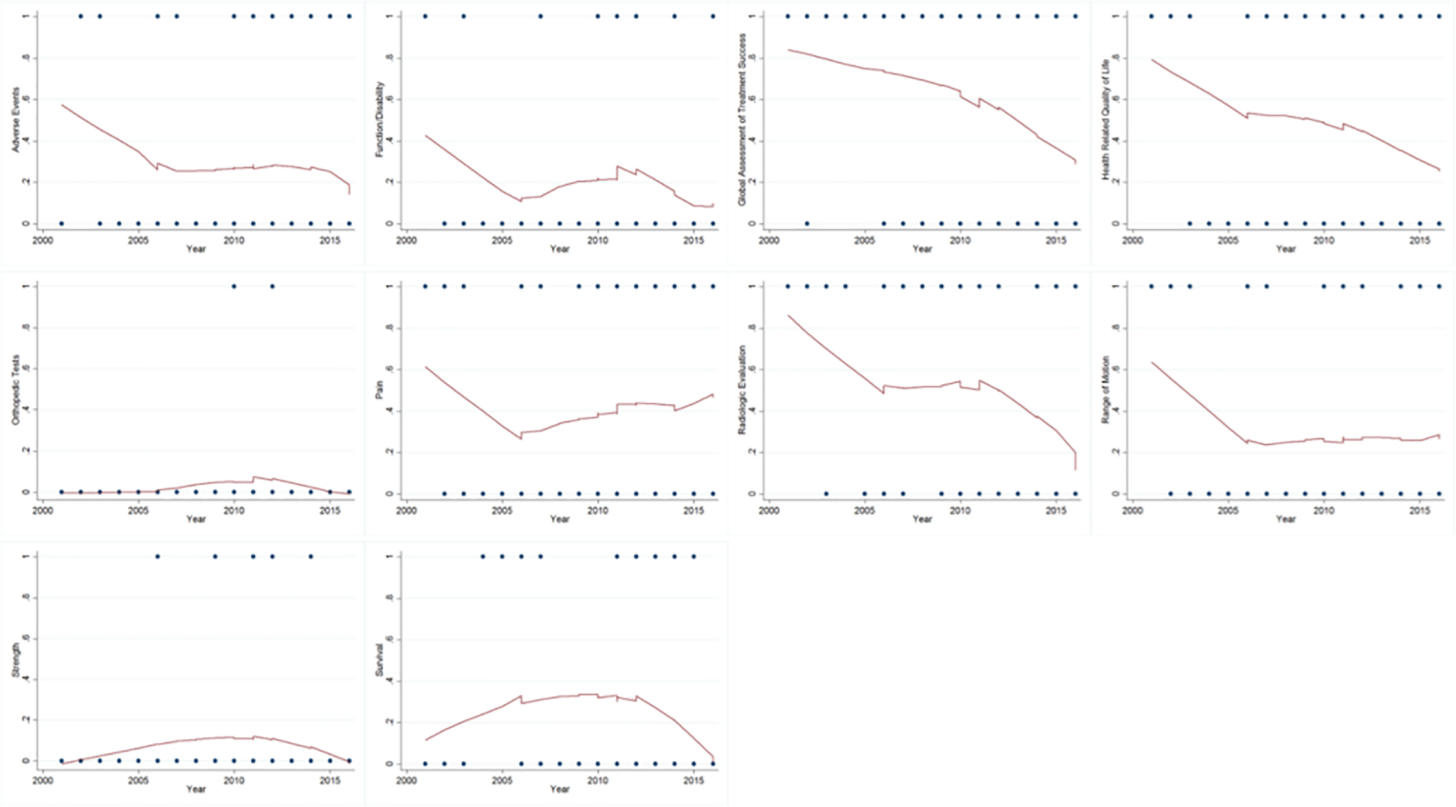
Smoothed scatterplots of outcome domain use over time. The frequency of reported outcomes over time are shown in these nonparametric regression locally weighted scatterplot smoothing method (LOWESS).

The frequency of reported outcomes over time is shown in Fig 2. Solid lines are smoothed values calculated from the nonparametric regression locally weighted scatterplot smoothing method (LOWESS). Visual inspection of the smoothed scatterplots indicates the survival outcome domain showed a trend of an overall increase from 2000 to 2016 while the pain outcome domain showed an increase following a significant decrease in reporting prior to 2005. The orthopedic tests and strength domains remained stable over time while global assessment of treatment success domain maintained a stable decline in outcome reporting over time (Fig 2).

## 4 Discussion

Results from our study suggest the need for greater standardization of outcomes as well as the instruments used to measure them. Interestingly, concurrent evaluations to ours by Page et al. [31–32] have affirmed the need for greater standardization of outcomes and measurement for shoulder disorders. Our findings are complimentary and confirmatory even though we used different search methodologies and applied different inclusion criteria. We limited our search to registered trials to minimize selective outcome reporting, whereas Page et al. reviewed published trials that served as primary studies in Cochrane reviews or were indexed in PubMed. Furthermore, while we examined outcomes reported across studies applying specific interventions (i.e., arthroplastic procedures), Page et al. looked more broadly at shoulder disorders. Despite these differences, we observed similar inconsistencies in trial outcomes. The lack of consistency observed in these studies indicates that developing a core outcome set for shoulder arthroplasty trials would be worthwhile. Such standardization would allow for more effective study to study comparisons in systematic reviews, while at the same time consider important outcomes that may be underrepresented otherwise.

While six outcomes, on average, were measured across trials, there were trials with as many as 37 outcomes measured in a single trial. Core outcome sets are developed to refine outcomes to those most meaningful and important across investigations and could help limit the number of outcomes being measured. Large numbers of outcomes in trials could result in increased occurrences of selective outcome reporting bias [33] or p-hacking [34], both of which may adversely affect our understanding of the true nature of clinical trial results.

We found a wide variety of shoulder instruments used across trials. For global assessment of treatment success, the Constant-Murley Score and ASES were used more frequently than other instruments. A systematic review of psychometric properties for the Constant-Murley Score reported the need for greater standardization for performing the score and greater caution during score interpretation [35]. Other issues, such as weighting the subscales, are ongoing matters of investigation with this scale. For most shoulder instruments, psychometric studies have focused on traditional validity and reliability estimates. Additional research is needed to determine important outcomes such as the minimal clinically important difference [35,36].

We noted several temporal trends in outcomes in this study. For example, our results suggest that HRQoL outcomes decreased over time. This finding is contrary to recent calls to include patient-centered outcomes in clinical research [37–41]. As early as 1990s, researchers recognized the importance of including patient-centered outcomes in orthopedic surgery research, rather than reliance on revision rates or clinical judgments to evaluate post-operative improvement [42]. Xu et al described HRQoL outcomes as a “necessity to fully understand the effects” of orthopedic interventions [43]. Furthermore, given recent indications of the prevalence of clinical depression in patients undergoing elective TSA, improved understanding of important quality of life variables is clearly warranted [44].

## 5 Limitations

Our study has the following limitations. We limited our sample to outcomes reported on ClinicalTrials.gov based on the recommendation of Clark and Williamson [45]. We chose this approach to include the most current outcomes, while simultaneously limiting selective outcome reporting bias. Although ClinicalTrials.gov is a United States based trial registry platform, there are currently 201 countries utilizing the registry and accounting for nearly 50% of registered studies [46]. Challenges also exist with registry-listed outcomes, which include the potential for vague and incomplete reporting. These challenges have been noted by the WHO and ClinicalTrials.gov, and actions are being taken to improve the accurate reporting of trial outcomes. We also did not search other trial registries, as Moja et al found that ClinicalTrials.gov contained enough data to adequately describe the ongoing research and was most valuable of all registries to finding ongoing clinical trials [47]. Furthermore, we wanted to avoid translating registrations that were written in other languages. We also did not search databases of published works, like MEDLINE or Embase, since published studies have been known to limit outcome reporting to only those which were found to be statistically significant [48–50]; therefore, the published literature may not contain all outcomes originally intended for measurement [51].

## 6 Conclusion

In summary, this study found a lack of standardization regarding outcomes and measurement devices. This lack of standardization limits systematic reviews to outcomes reported and measured consistently across studies. Important outcomes may be omitted from a subset of studies, limiting data synthesis. Our study provides a summary of outcomes most frequently reported and co-occurring outcomes as a foundation for a follow up study to begin developing a core outcome set for shoulder arthroplasty studies.

## Supporting information

S1 File PRISMA checklist. (DOC)

## References

[1] Killian ML, Cavinatto L, Galatz LM, Thomopoulos S. Recent advances in shoulder research. *Arthritis Res & Ther*. 2012;14 doi: 10.1186/ar3846 22709417 10.1186/ar3846 PMC3446497

[2] Day JS, Lau E, Ong KL, Williams GR, Ramsey ML, Kurtz SM. Prevalence and projections of total shoulder and elbow arthroplasty in the United States to 2015. *J Should Elbow Surg*. 2010;19:1115–1120. doi: 10.1016/j.jse.2010.02.009 20554454 10.1016/j.jse.2010.02.009

[3] Izquierdo R, Voloshin I, Edwards S, Freehill MQ, Stanwood W, Wiater JM, et al Treatment of glenohumeral osteoarthritis. *J Am Acad Orthop Surg*. 2010;18:375–382. doi: 10.5435/00124635-201006000-00010 20511443 10.5435/00124635-201006000-00010

[4] Wiater JM, Fabing MH. Shoulder arthroplasty: prosthetic options and indications. *J Am Acad Orthop Surg*. 2009;17:415–425. doi: 10.5435/00124635-200907000-00002 19571297 10.5435/00124635-200907000-00002

[5] Bohsali KI, Wirth MA, Rockwood CA. Complications of total shoulder arthroplasty. *J Bone Joint Surg*. *Am*. 2006;88:2279–2292. doi: 10.2106/JBJS.F.00125 17015609 10.2106/JBJS.F.00125

[6] Cheung EV, Diaz R, Athwal GS, Sanchez-Sotelo J, Sperling JW. Shoulder Arthroplasty: Key Steps to Improve Outcomes and Minimize Complications. *Instr Course Lect*. 2015;65:109–126. 27049185

[7] Cvetanovich GL, Frank RM, Chalmers PN, Verma NN, Nicholson GP, Romeo AA. Surgical Management of Proximal Humeral Fractures: The Emerging Role of Reverse Total Shoulder Arthroplasty. *Orthopedics*. 2016;39:e465–e473. doi: 10.3928/01477447-20160324-02 27045483 10.3928/01477447-20160324-02

[8] Singh JA, Sperling J, Buchbinder R, McMaken K. Surgery for shoulder osteoarthritis. *Cochrane Database Syst Rev*. 2010;(10). doi: 10.1002/14651858.CD008089.pub2 20927773 10.1002/14651858.CD008089.pub2

[9] Horner NS, de Sa D, Heaven S, Simunovic N, Bedi A, Athwal GS, et al Indications and outcomes of shoulder arthroscopy after shoulder arthroplasty. *J Should Elbow Surg*. 2016;25:510–518. doi: 10.1016/j.jse.2015.09.013 26652703 10.1016/j.jse.2015.09.013

[10] Clarke M, Williamson PR. Core outcome sets and systematic reviews. *Syst Rev*. 2016;5 doi: 10.1186/s13643-016-0188-6 26792080 10.1186/s13643-016-0188-6 PMC4719739

[11] Harman NL, Bruce IA, Kirkham JJ, Tierney S, Callery P, O’Brien K, et al The Importance of Integration of Stakeholder Views in Core Outcome Set Development: Otitis Media with Effusion in Children with Cleft Palate. *PloS one*. 2015;10 doi: 10.1371/journal.pone.0129514 26115172 10.1371/journal.pone.0129514 PMC4483230

[12] Tunis SR, Clarke M, Gorst SL, Gargon E, Blazeby JM, Altman DG, et al Improving the relevance and consistency of outcomes in comparative effectiveness research. *J Comp Eff Res*. 2016;5:193–205. doi: 10.2217/cer-2015-0007 26930385 10.2217/cer-2015-0007 PMC4926524

[13] Boers M, Kirwan JR, Tugwell P, Beaton D, Bingham III CO, Conaghan PG, et al The OMERACT Handbook OMERACT 2014 Available at http://www.omeract.org/pdf/OMERACT_Handbook.pdf. Accessed June 21, 2016.

[14] Angst F, Schwyzer HK, Aeschlimann A, Simmen BR, Goldhahn J. Measures of adult shoulder function: Disabilities of the Arm, Shoulder, and Hand Questionnaire (DASH) and its short version (QuickDASH), Shoulder Pain and Disability Index (SPADI), American Shoulder and Elbow Surgeons (ASES) Society standardized shoulder assessment form, Constant (Murley) Score (CS), Simple Shoulder Test (SST), Oxford Shoulder Score (OSS), Shoulder Disability Questionnaire (SDQ), and Western Ontario Shoulder Instability Index (WOSI). *Arthritis Care & Res*. 2011;63 Suppl 11:S174–S188. doi: 10.1002/acr.20630 22588743 10.1002/acr.20630

[15] Wright RW, Baumgarten KM. Shoulder outcomes measures. *J Am Acad Orthop Surg*. 2010;18:436–444. doi: 10.5435/00124635-201007000-00006 20595136 10.5435/00124635-201007000-00006

[16] Roe Y, Soberg HL, Bautz-Holter E, Ostensjo S. A systematic review of measures of shoulder pain and functioning using the International classification of functioning, disability and health (ICF). *BMC Musculoskelet Disord*. 2013;14 doi: 10.1186/1471-2474-14-73 23445557 10.1186/1471-2474-14-73 PMC3668165

[17] 45 CFR 46 | HHS.gov. Available at http://www.hhs.gov/ohrp/regulations-and-policy/regulations/45-cfr-46/. Published June 21, 2016. Accessed June 21, 2016.

[18] Li T, Vedula SS, Hadar N, Parkin C, Lau J, Dickersin K. Innovations in data collection, management, and archiving for systematic reviews. *Ann Intern Med*. 0;162:287–294. doi: 10.7326/M14-1603 25686168 10.7326/M14-1603

[19] Cochrane Handbook for Systematic Reviews of Interventions. Available at http://handbook.cochrane.org/. Published November 19, 2014. Accessed June 21, 2016.

[20] Standards for Systematic Reviews: Health and Medicine Division. Available at http://www.nationalacademies.org/hmd/Reports/2011/Finding-What-Works-in-Health-Care-Standards-for-Systematic-Reviews/Standards.aspx. Accessed June 21, 2016.

[21] PRISMA 2009 Checklist. Available at http://www.prisma-statement.org/documents/PRISMA2009checklist.pdf. Accessed June 22, 2016.

[22] Lang TA, Altman DG. Basic statistical reporting for articles published in biomedical journals: The “Statistical Analyses and Methods in the Published Literature” or The SAMPL Guidelines In: Smart P, Maisonneuve H, Polderman A (eds). *Science Editors’ Handbook*, European Association of Science Editors, 2013 Available at http://www.equator-network.org/wp-content/uploads/2013/07/SAMPL-Guidelines-6-27-13.pdf. Accessed June 21, 2016. 10.1016/j.ijnurstu.2014.09.006 25441757

[23] Williams RJ, Tse T, Harlan WR, Zarin DA. Registration of observational studies: is it time? *CMAJ*. 2010;182:1638–1642. doi: 10.1503/cmaj.092252 20643833 10.1503/cmaj.092252 PMC2952011

[24] Glossary, CRG, NHLBI, NIH. Available at http://www.nhlbi.nih.gov/research/funding/research-support/crg/application/observational-interventional.htm. Published June 29, 2016. Accessed June 29, 2016.

[25] Benstoem C, Moza A, Autschbach R, Stoppe C, Goetzenich A. Evaluating outcomes used in cardiothoracic surgery interventional research: a systematic review of reviews to develop a core outcome set. *PloS one*. 2015;10 doi: 10.1371/journal.pone.0122204 25830921 10.1371/journal.pone.0122204 PMC4382223

[26] Hopkins JC, Howes N, Chalmers K, Savovic J, Whale K, Coulman KD, et al Outcome reporting in bariatric surgery: an in-depth analysis to inform the development of a core outcome set, the BARIACT Study. *Obes Rev*. 2015;16:88–106. doi: 10.1111/obr.12240 25442513 10.1111/obr.12240

[27] McNair AGK, Whistance RN, Forsythe RO, Rees J, Jones JE, Pullyblank AM, et al Synthesis and summary of patient-reported outcome measures to inform the development of a core outcome set in colorectal cancer surgery. *Colorectal Dis*. 2015;17:O217–O229. doi: 10.1111/codi.13021 26058878 10.1111/codi.13021 PMC4744711

[28] Page MJ, McKenzie JE, Green SE, Beaton DE, Jain NB, Lenza M, et al Core domain and outcome measurement sets for shoulder pain trials are needed: systematic review of physical therapy trials. *J Clin Epidemiol*. 2015;68:1270–1281. doi: 10.1016/j.jclinepi.2015.06.006 26092288 10.1016/j.jclinepi.2015.06.006 PMC4711903

[29] Saldanha IJ, Dickersin K, Wang X, Li T. Outcomes in Cochrane systematic reviews addressing four common eye conditions: an evaluation of completeness and comparability. *PloS one*. 2014;9 doi: 10.1371/journal.pone.0109400 25329377 10.1371/journal.pone.0109400 PMC4199623

[30] Whitehead L, Perkins GD, Clarey A, Haywood KL. A systematic review of the outcomes reported in cardiac arrest clinical trials: the need for a core outcome set. *Resuscitation*. 2015;88:150–157. doi: 10.1016/j.resuscitation.2014.11.013 25497393 10.1016/j.resuscitation.2014.11.013

[31] Buchbinder R, Page MJ, Huang H, Verhagen AP, Beaton D, Kopkow C, et al A Preliminary Core Domain Set for Clinical Trials of Shoulder Disorders: A Report from the OMERACT 2016 Shoulder Core Outcome Set Special Interest Group. J Rheumatol. 2017; doi: 10.3899/jrheum.161123 28089972 10.3899/jrheum.161123

[32] Page MJ, Huang H, Verhagen AP, Gagnier JJ, Buchbinder R. Outcome reporting in randomized trials for shoulder disorders: Literature review to inform the development of a core outcome set. Arthritis Care Res. 2017; doi: 10.1002/acr.23254 28388821 10.1002/acr.23254

[33] Rongen JJ, Hannink G. Comparison of Registered and Published Primary Outcomes in Randomized Controlled Trials of Orthopaedic Surgical Interventions. *J Bone Joint Surg Am*. 2016;98:403–409. doi: 10.2106/JBJS.15.00400 26935463 10.2106/JBJS.15.00400

[34] Head ML, Holman L, Lanfear R, Kahn AT, Jennions MD. The extent and consequences of p-hacking in science. *PLoS Biol*. 2010;13 doi: 10.1371/journal.pbio.1002106 25768323 10.1371/journal.pbio.1002106 PMC4359000

[35] Roy JS, MacDermid JC, Woodhouse LJ. A systematic review of the psychometric properties of the Constant-Murley score. *J Shoulder Elbow Surg*. 2010;19:157–164. doi: 10.1016/j.jse.2009.04.008 19559630 10.1016/j.jse.2009.04.008

[36] Roy JS, MacDermid JC, Woodhouse LJ. Measuring shoulder function: a systematic review of four questionnaires. *Arthritis Rheum*. 2009;61:623–632. doi: 10.1002/art.24396 19405008 10.1002/art.24396

[37] Chau DB, Ciullo SS, Watson-Smith D, Chun TH, Kurkchubasche AG, Luks FI. Patient-centered outcomes research in appendicitis in children: Bridging the knowledge gap. *J Pediatr Surg*. 2016;51:117–121. doi: 10.1016/j.jpedsurg.2015.10.029 26545589 10.1016/j.jpedsurg.2015.10.029

[38] Kanzaria HK, McCabe AM, Meisel ZM, LeBlanc A, Schaffer JT, Bellolio MF, et al Advancing Patient-centered Outcomes in Emergency Diagnostic Imaging: A Research Agenda. *Acad Emerg Med*. 2015;22:1435–1446. doi: 10.1111/acem.12832 26574729 10.1111/acem.12832

[39] Mancuso CA, Duculan R, Cammisa FP, Sama AA, Hughes AP, Lebl DR, et al Proportion of Expectations Fulfilled: A New Method to Report Patient-centered Outcomes of Spine Surgery. *Spine*. 2016;41:963–970. doi: 10.1097/BRS.0000000000001378 26679871 10.1097/BRS.0000000000001378

[40] Rising KL, Carr BG, Hess EP, Meisel ZF, Ranney ML, Vogel JA. Patient-centered Outcomes Research in Emergency Care: Opportunities, Challenges, and Future Directions. *Acad Emerg Med*. 2016;23:497–502. doi: 10.1111/acem.12944 26919027 10.1111/acem.12944 PMC5222628

[41] Zygmont ME, Lam DL, Nowitzki KM, Burton KR, Lenchik L, McArthur TA, et al Opportunities for Patient-centered Outcomes Research in Radiology. *Acad Radiol*. 2016;23:8–17. doi: 10.1016/j.acra.2015.08.027 26683507 10.1016/j.acra.2015.08.027

[42] Beaton DE, Bombardier C, Katz JN, Wright JG, Wells G, Boers M, et al Looking for important change/differences in studies of responsiveness. OMERACT MCID Working Group. Outcome Measures in Rheumatology. Minimal Clinically Important Difference. *J Rheumatol*. 2001;28:400–405. 11246687

[43] Xu M, Garbuz DS, Kuramoto L, Sobolev B. Classifying health-related quality of life outcomes of total hip arthroplasty. *BMC Musculoskelet Disord*. 2005;6 doi: 10.1186/1471-2474-6-48 16144550 10.1186/1471-2474-6-48 PMC1242235

[44] Mollon B, Mahure SA, Ding DY, Zuckerman JD, Kwon YW. The influence of a history of clinical depression on peri-operative outcomes in elective total shoulder arthroplasty: a ten-year national analysis. *Bone Joint J*. 2016;98-B:818–824. doi: 10.1302/0301-620X.98B6.37208 27235526 10.1302/0301-620X.98B6.37208

[45] Clarke M, Williamson P: Core outcome sets and trial registries. Trials 2015; 16:216 doi: 10.1186/s13063-015-0738-6 25971905 10.1186/s13063-015-0738-6 PMC4446826

[46] Trends, Charts, and Maps. ClinicalTrials.gov. Available at https://clinicaltrials.gov/ct2/resources/trends. Accessed on October 11, 2017.

[47] Moja LP, Moschetti I, Nurbhai M, et al Compliance of clinical trial registries with the World Health Organiztion minimum data set: a survey. Trials [Electronic Resource] 2009; 10:56. 10.1186/1745-6215-10-56 PMC2734552 19624821

[48] Nissen T, Wayant C, Wahlstrom A, Sinnett P, Fugate C, Herrington J, Vassar M. Methodological quality, completeness of reporting and use of systematic reviews as evidence in clinical practice guidelines for paediatric overweight and obesity. Clin Obes 2017; 2017;7(1):34–45. doi: 10.1111/cob.12174 28112500 10.1111/cob.12174

[49] Howard B, Scott JT, Blubaugh M, Roepke B, Scheckel C, Vassar M. Systematic review: Outcome reporting bias is a problem in high impact factor neurology journals. PLoS One 2017;12(7):e0180986 doi: 10.1371/journal.pone.0180986 28727834 10.1371/journal.pone.0180986 PMC5519049

[50] Wayant C, Scheckel C, Hicks C, Nissen T, Leduc L, Som M, Vassar M. Evidence of selective reporting bias in hematology journals: A systematic review. PLoS One 2017;12(6):e0178379 doi: 10.1371/journal.pone.0178379 28570573 10.1371/journal.pone.0178379 PMC5453439

[51] Fleming PS, Koletsi D, Dwan K, Pandis N. Outcome discrepancies and selective reporting: impacting the leading journals? PLoS ONE. 2015; 10(5):e0127495 doi: 10.1371/journal.pone.0127495 ; PubMed Central PMCID: PMC4440809. 25996928 10.1371/journal.pone.0127495 PMC4440809

